# Exploring risk factors for pediatric cancer patients admitted to the Pediatric Intensive Care Unit: insight from a multicenter observational study revealing no association with mechanical ventilation

**DOI:** 10.1186/s44158-025-00275-6

**Published:** 2025-10-14

**Authors:** Angela Amigoni, Sara Boscato, Maria Cristina Mondardini, Francesca Cavagnero, Luca Marchetto, Veronica Biassoni, Carolina Birolo, Gabriella Bottari, Manuela Corno, Stefania Ferrario, Giorgia Maiolo, Alessia Montaguti, Emanuele Rossetti, Immacolata Rulli, Raffaella Sagredini, Stefania Spaggiari, Luisa Vatiero, Gianluca Vigna, Matteo Martinato, Dario Gregori, Marta Pillon, Rosanna Irene Comoretto, OncoTIPNet Study Group, Luca Arcuri, Elena Barisone, Massimo Bellettato, Paolo Biban, Alessandra Biffi, Arcangelo Prete, Ezio Bonanomi, Fabio Caramelli, Corrado Cecchetti, Alessandra Conio, Emanuele Guacheri, Alberto Giannini, Andrea Moscatelli, Sergio Picardo, Zaccaria Ricci, Elena Zoia

**Affiliations:** 1 Pediatric Intensive Care Unit, Department of Women’s and Children’s Health, University Hospital of Padua, Padua, Italy; 2 University of Padua, Padua, Italy; 3 Pediatric Intensive Care Unit, IRCCS AOUBO, Via Albertoni 15, Bologna, 40138 Italy; 4 Pediatric Oncologic Unit, Fondazione IRCCS, Istituto Nazionale Dei Tumori, Milan, Italy; 5 S. Bortolo Hospital, Vicenza, Italy; 6 DEA Unit, Bambino Gesù Chidren’s Hospital, Rome, Italy; 7 Papa Giovanni, XXIII Hospital, Bergamo, Italy; 8 Buzzi Children’s Hospital, Milan, Italy; 9 AOU Città Della Salute E Della Scienza, Regina Margherita Hospital, Turin, Italy; 10 Neonatal and Pediatric Intensive Care Unit, Emergency Department, IRCCS Giannina Gaslini Children’s Hospital, Genoa, Italy; 11 ARCO Unit, Bambino Gesù Children’s Hospital, Rome, Italy; 12 Terapia Intensiva Neonatale E Pediatrica, University Hospital Policlinico G. Martino, Messina, Italy; 13 Burlo Garofalo Hospital, Trieste, Italy; 14 University Hospital of Verona, Verona, Italy; 15 Meyer Children’s Hospital, Florence, Italy; 16 Spedali Civili, Brescia, Italy; 17 Unit of Biostatistics, Epidemiology and Public Health, Department of Cardiac, Thoracic, Vascular Sciences and Public Health, University of Padua, Padua, Italy; 18 Pediatric Oncologic Unit, University Hospital of Padua, Padua, Italy; 19 Department of Sciences of Public Health and Pediatrics, University of Turin, Turin, Italy

**Keywords:** Intensive care units, Pediatric, Cancer, Risk factors, Outcomes

## Abstract

**Background:**

To analyze risk factors for adverse outcomes in a nationally representative sample of pediatric cancer patients admitted to the PICU.

**Methods:**

An observational study composed of a 2-year retrospective phase and a 2-year prospective phase was conducted before and during PICU admission in Italian PICUs.

**Results:**

We included 518 patients, median age 7.2 years (IQR 2.5–12.6). Main diagnosis: solid tumors (51%) and acute lymphoblastic leukemia (23%). Nineteen percent underwent stem cell transplantation (HSCT). Main causes of admission were respiratory failure (33%) and neurological impairment (24%). In-PICU mortality was 15%, higher in HSCT (41%) and non-solid cancer (25%). Pre-PICU mortality risk factors included HSCT (OR 3.48, 95%CI 1.5–8.11), higher Pediatric Overall Performance Category (POPC) (OR 1.72, 95%CI 1.23–2.42), and Pediatric Index of Mortality 3 (PIM-3) score (OR 1.03, 95%CI 1.01–1.06). In-PICU mortality risk factors included multiple organ failure (MOF) (OR 4.83, 95%CI 1.66–15.71), and cardiac arrest (OR 82.16, 95%CI 14.19–1594.61). The use of MV does not appear to be associated with increased mortality. Longer PICU LOS was associated with pre-admission acute respiratory distress syndrome (*p* < 0.001), renal failure (*p* = 0.024), POPC (*p* = 0.007) and PIM 3 (*p* < 0.001), and in-PICU use of total parenteral nutrition (*p* = 0.036), and duration of mechanical ventilation (MV) (*p* < 0.001).

**Conclusions:**

HSCT, non-solid tumor, higher PIM-3, and POPC on admission, MOF, and history of cardiac arrest were associated with poorer outcome. The use of MV does not appear to be associated with increased mortality.

**Trial registration:**

ClinicalTrials.gov ID NCT04581655, October 7, 2020.

## Background

Over the last decades, significant improvements have been made in pediatric oncology, due to more effective treatment protocols, a deeper understanding of cancer biology, and advancements in Hematopoietic Stem Cell Transplantation (HSCT), leading to improved outcomes for pediatric cancer patients. However, pediatric cancer patients may still require admission to the PICU during oncologic treatment due to the complexity of the underlying disease or treatment-related complications.

While survival rates have recently improved [1–5], mortality rates remain higher (about 30% to 40%) compared to the general PICU population [5, 6].

HSCT recipients admitted to the PICU have been studied extensively, with research suggesting they face an overall higher risk of complications compared to non-transplanted patients, resulting in higher mortality rates [7–10]. Previous studies, albeit analyzing rather small population samples, have also identified other mortality risk factors in pediatric cancer patients admitted to the PICU: multiple organ failure (MOF), sepsis, mechanical ventilation (MV), inotropic support, acute kidney injury (AKI), and the Continuous Renal Replacement Therapy (CRRT) [1–3, 7–18].

The objective of this multicenter study was to analyze risk factors associated with mortality and PICU Length of Stay (LOS) in a pediatric cancer population sample prospectively defined.

## Methods

### Design and setting of the study

This was an observational multicenter ambispective cohort study on oncological patients admitted to the PICU. It involved 14 Italian PICUs and consisted of a retrospective and a prospective phase. The retrospective phase data were obtained by reviewing the medical records of pediatric cancer patients. The prospective data were registered on a web-based database (REDCAP). Study approval was granted by the Ethics Committee of the promoting center (University Hospital of Padova) on June 17th, 2021 (Protocol No. CESC 5068/AO/21) and by all Research Ethics Committees at participating sites. The protocol was prospectively registered on ClinicalTrials.gov (ID NCT04581655). Parental written informed consent was obtained prior to enrollment.

This study complies with the Strengthening the Reporting of Observational Studies in Epidemiology (STROBE) guidelines, and is endorsed by the Italian Society of Pediatric and Neonatal Anesthesia and Critical Care (SARNEPI) and the Italian Association of Hematology and Pediatric Oncology (AIEOP).

### Study population

Data were collected from patients with the following inclusion criteria: age < 18 years, diagnosis of/ongoing treatment for oncologic disease, admission to the PICU. The exclusion criteria were: informed consent not obtained and medical record access not available.

Sample size was estimated using a precision-based approach for estimating binomial confidence intervals with in-PICU mortality as the primary endpoint. A mortality rate of 30% [1] with a 95% confidence interval (CI) and a margin of error of 4% was assumed from the literature. Based on these parameters, the required sample size for the study is 505 subjects.

### Data collection and outcomes

Data collected through the REDCap platform [19, 20] of the Unit of Biostatistics, Epidemiology and Public Health of the University of Padova were related to:

1. *Patients’ characteristics and variables before PICU admission:* sex, age, oncological disease and corresponding treatment phase, history of and type of HSCT, advanced treatments received before PICU admission (high-flow nasal cannula [HFNC], vasoactive treatment non-invasive ventilation [NIV], dialysis), presence of MOF and pediatric Acute Respiratory Distress Syndrome (PARDS), Pediatric Overall Performance Category (POPC) score;
2. *Variables collected during PICU stay:* unit/hospital before PICU, type and reason for PICU admission, Pediatric Index of Mortality (PIM) 3 score, Pediatric Logistic Organ Dysfunction-2 (PELOD-2) score, priority levels for PICU admission, presence of organ failure, MOF, infection and sepsis, episodes of cardiac arrest. Total parenteral nutrition (TPN), dialysis, invasive (InV) and non-invasive (NIV) ventilation (before or after intubation), Extracorporeal Membrane Oxygenation (ECMO), POPC at PICU discharge.

The primary outcome of the study was in-PICU mortality, and the secondary outcome was PICU LOS.

### Definitions

Priority levels definition (listed below) was created by the TIPNET group Italian PICUs to identify the patient’s need and limitation of care for PICU admission.

- *Level 1*: Critical, unstable patients requiring intensive care and monitoring; no limitation of care;
- *Level 2*: Patients requiring intensive monitoring and possibly immediate treatment; no limitation of care;
- *Level 3*: Critical, unstable patients with low expectation of recovery; limited care;
- *Level 4*: Patients not suitable for PICU (4a: patients who may not benefit from PICU admission, treatment may be provided in non-intensive care units; 4b: patients with terminal illness at high risk of mortality).

Organ failure (listed below) was defined as reported in the TIPNET database, after approval by National consensus conference available at www.tipnet.org:

- *Respiratory failure* *: *need for oxygen therapy or MV;
- *Neurological failure* *: *Glasgow Coma Scale score < 8;
- *Hemodynamic failure*: need for vasoactive agents;
- *Hepatic failure*: total plasma bilirubin > 4 mg/dl in the absence of hemolysis or liver disease; ALT levels greater than 2 times the upper age limit;
- *Acute Kidney Injury*: oliguria or anuria, renal impairment (KDIGO) grade 2 or higher [21];
- MOF: concurrent failure of 2 or more organs.
- PARDS: if present at PICU admission or developed during PICU stay, was defined according to PALICC-2 [22].
- Sepsis: was defined as confirmed or suspected infection and an acute increase in pSOFA score of 2 points or more from up to 48 h before infection to 24 h after infection and receiving antimicrobial therapy [23].
- PIM-3: was calculated within the first hour after PICU admission [24],
- PELOD-2 score: was calculated on day 1, day 2 and at discharge [25].

### Statistical analysis

A descriptive analysis of the sample was reported using median and interquartile range for continuous variables and absolute numbers and relative percentages for categorical ones. The study sample was then described according to the procedure of HSCT and the diagnosis of solid (vs hematological) tumor. The presence of statistically significant differences between groups was assessed using the Kruskal–Wallis test for continuous variables and the chi-squared test for categorical ones, after adjusting the test values for test multiplicity according to the method by Benjamini & Hochberg [26].

Univariate and multivariate logistic were used to assess the association between patients’ characteristics and the primary outcome (in-PICU mortality). For the secondary outcome (PICU LOS), which includes some zero values, a Zero-Adjusted Gamma (ZAGA) regression model was implemented [27]. This approach accounts for the presence of exact zeros while modeling the positive observations with a Gamma distribution. The results are presented in terms of Exp(μ), which represents the multiplicative effect on the expected outcome. Specifically, (Exp(μ)—1) × 100% indicates the percentage change in the expected outcome for a one-unit increase in the predictor. Bootstrap resampling (1,000 iterations) was used to estimate 95% CI. Model fit was assessed using Akaike’s Information Criterion (AIC) and residual diagnostics.

The models considered both pre-PICU and in-PICU patient characteristics. For multivariable regression models, the best models were selected based on the a priori clinical relevance of the variables and the goodness of fit of each model as calculated by the R^2^ index or the AIC (Akaike information criterion; the lowest AIC indicates that the model best fits the data). All statistical tests were two-sided, and the level of statistical significance was set at 0.05. Data were analyzed using the R software version 4.3.0 [28] and *gamlss* package.

## Results

Investigators performed a retrospective (2019–2021) and a prospective analysis (2021–2022).

### Patient characteristics pre PICU admission

A total of 518 patients were included in the study (236 and 282 patients in the retrospective and prospective phase, respectively). Ten patients were excluded from the retrospective phase because informed consent could not be obtained, and 24 were excluded because access to their medical records was unavailable. Fifty-four percent of the sample were male and the median age was 7.2 years (IQR 2.5–12.6). Prior to PICU admission, 98 patients (32%) had MOF and 42 (19%) had PARDS. Pre-admission POPC scores were in the normal or mild disability categories (≤ 2) in 78% of the sample (Table 1). Prior to PICU admission, advanced treatments were reported in 164 (32%) patients, mainly HFNC oxygenation (44%), followed by dopamine infusion (16%), NIV (14%), and renal replacement therapies (hemofiltration and dialysis, each at 2%).

**Table 1:** Patient demographics and clinical characteristics

Characteristic	All sample *N* = 518
Sex (male)	280 (54%)
Age (years) missing	7.2 (2.5—12.6) 7 (1.35%)
Type of disease	
Solid tumor	262 (51%)
ALL	119 (23%)
AML	32 (6.2%)
NHL	20 (3.9%)
HL	6 (1.2%)
Other Missing	75 (15%) 4 (0.77%)
Treatment phase	
On therapy	233 (46%)
Out of therapy	91 (18%)
Not yet started therapy Missing	185 (36%) 9 (1.74%)
HSCT missing	95 (19%) 6 (11.16%)
Type of HSCT	
Family donor allogeneic transplantation	51 (54%)
Autologous	24 (26%)
Family donor haploidentical transplantation Missing	19 (20%) 1 (0.19%)
MOF (before PICU admission) Missing	98 (32%) 213 (41.12%)
PARDS (before PICU admission) Missing	42 (19%) 292 (56.37%)
POPC	
1	213 (43%)
2	173 (35%)
3	48 (9.7%)
4	53 (11%)
5 Missing	6 (1.2%) 25 (4.83%

Data are presented using frequencies and percentages for categorical variables and medians and interquartile range (IQR) for continuous ones

*Abbreviations*: *OE* Onco-haematological, *ALL* Acute lymphoblastic leukaemia, *AML* Acute myeloid leukaemia, *NHL* Non-Hodgkin lymphoma, *HL* Hodgkin lymphoma, *HSCT* Hematopoietic stem-cell transplantation, *MOF* Multiple organ failure, *PICU* Paediatric intensive care unit, *PARDS* Paediatric acute respiratory distress syndrome, *POPC* Paediatric overall performance category

### Patient characteristics at PICU admission

Most patients were transferred from another unit in the same hospital (61%). The priority level was mainly 1 (56%) or 2 (37%). Patients were admitted to the PICU mainly for medical reasons (59%): respiratory failure (33%) and neurological failure (24%). Ninety-five patients (18%) were admitted to the PICU after surgery with a LOS of less than 48 h. The median PIM3 on admission was 2% (IQR 1–8%) and the median PELOD-2 score was 0.6 (IQR 0.1–1.4) (Table 2).

**Table 2:** Patients’ characteristics at PICU admission

Characteristic	All sample *N *= 518
Admission from	
Unit of the same hospital	314 (61%)
Unit of other hospitals	69 (14%)
Emergency department	44 (8.6%)
PICU of other hospitals	11 (2.2%)
Other Missing	73 (14%) 7 (1.35%)
Priority of admission	
1	279 (56%)
2	184 (37%)
3	26 (5.3%)
4a	3 (0.6%)
4b Missing	3 (0.6%) 23 (4.44%)
Type of admission	
Medical	300 (59%)
Surgical Missing	206 (41%) 12 (2.32%)
Surgical patients with LOS less than 48 h	95 (18%)
Reason of admission	
Respiratory failure	95 (33%)
Neurological failure	70 (24%)
Hemodynamic failure	25 (8.5%)
Hepatic failure	9 (3.1%)
Acute Kidney Injury	6 (2.0%)
Other Missing	89 (30%) 224 (43.24%)
PIM3 at PICU admission Missing	2 (1, 8) 60 (11.59%)
PELOD-2 score at PICU admission Missing	0.6 (0.1, 1.4) 104 (20.08%)

Data are presented using frequencies and percentages for categorical variables and medians and interquartile range (IQR) for continuous ones

*Abbreviations*: *PICU* Paediatric intensive care unit, *LOS* Length of stay, *PIM* Paediatric index of mortality expressed as %, *PELOD* Paediatric logistic organ dysfunction

### Patient characteristics during PICU stay

During PICU stay, MOF was reported in 46%, respiratory failure in 35%, hemodynamic failure in 17%, and renal acute injury in 12% of the sample (Table 3). Infection was recorded in 165 patients (32%) of which 64 (38% of infections, 12.3% of total sample) progressed to sepsis. The median PELOD-2 score on day 2 was 0.3 (IQR 0.1–1.4) and 0 (IQR 0.0–1.0) on the day of discharge. Thirty-four patients (7%) experienced cardiac arrest during the PICU stay. 266 patients (57%) were mechanically ventilated, 97 (21%) with NIV (65% of them before intubation) and 233 (50%) with InV. TPN was administered to 156 patients (33%). In-PICU mortality was 15% in the total sample.

**Table 3:** Patients’ in-PICU characteristics, treatments and outcomes

Characteristic	All sample *N* = 518
PELOD-2 score on the second day missing	0.3 (0.1, 1.4) 213 (41.12%)
PELOD-2 score at PICU discharge missing	0 (0, 1) 104 (20.08%)
MOF (during PICU admission) missing	99 (46%) 301 (58.1%)
Respiratory failure	183 (35%)
Hemodynamic failure	86 (17%)
Acute Kidney Injury	64 (12%)
Infection Sepsis	165 (32%) 64 (12.3%)
Cardiac arrest (missing 7.33%)	34 (7%)
Dialysis	39 (7.5%)
ECMO	3 (0.6%)
TPN (missing 7.33%)	156 (32%)
Mechanical Ventilation (missing 10.42%)	266 (57%)
NIV	97 (21%)
NIV pre-IV	63 (65%)
IV	233 (50%)
NIV post-IV	47 (48%)
LOS (days) (missing 2.32%)	3 (1—9)
Mortality (missing 2.12%)	
In-PICU mortality	78 (15%)
30-days mortality	8 (1.6%)
90-days mortality	7 (1.4%)
POPC at PICU discharge (missing 21.62%)	
1	152 (37%)
2	147 (36%)
3	69 (17%)
4	33 (8.1%)
5	5 (1.2%)

Data are presented using frequencies and percentages for categorical variables and medians and interquartile range (IQR) for continuous ones

*Abbreviations*: *PICU* Paediatric intensive care unit, *LOS* Length of stay, *PIM* Paediatric index of mortality, *PELOD* Paediatric logistic organ dysfunction, *MOF* Multiple organ failure, *ECMO* Extracorporeal membrane oxygenation, *TPN* Total parenteral nutrition, *NIV* Non-invasive ventilation, *IV* Invasive ventilation, *POPC* Paediatric overall performance category

### HSCT versus non-HSCT patients

Comparing patients who underwent HSCT with those who did not (Table 4), HSCT patients were more likely to be slightly older (*p* = 0.049), to be admitted from the same hospital unit (*p* = 0.001), and to be in the treatment phase of the disease (*p* < 0.001), POPC was higher (*p* = 0.005). Prior to PICU admission, HSCT patients were more likely to have received advanced treatments (*p* < 0.001), to have developed MOF and PARDS (*p* < 0.001). During their stay in the PICU, patients more frequently developed MOF (*p* = 0.005), TPN use was more frequent (*p* < 0.001), MV was longer (*p *< 0.001), cardiac arrest (*p* < 0.001) and infections (*p* < 0.001) were more frequent. HSCT patients had higher PELOD-2 (*p* < 0.001) and PIM-3 (*p* < 0.001) scores and in-PICU mortality rate (41% vs 10%, *p* < 0.001).

**Table 4:** Sample characteristics according to HSCT (HSCT/no HSCT)

**Characteristic**	***N***	**HSCT**, *N* = 95	**No HSCT**, *N* = 417	**Overall**, *N* = 512	***p*-value**
Age (years) Treatment phase	510 508	9.0 (4.0–13.7)	6.9 (2.2–12.3)	7.2 (2.5–12.6	0.049 <0.001
On therapy		49 (53%)	184 (44%)	233 (46%)	
Out of therapy		37 (40%)	54 (13%)	91 (18%)	
Not yet started therapy		7 (7.5%)	177 (43%)	184 (36%)	
Admission from	511				0.001
Unit of the same hospital		71 (75%)	243 (58%)	314 (61%)	
Unit of other hospitals		9 (9.5%)	60 (14%)	69 (14%)	
Emergency department		4 (4.2%)	40 (9.6%)	44 (8.6%)	
PICU of other hospitals		4 (4.2%)	7 (1.7%)	11 (2.2%)	
Other		7 (7.4%)	66 (16%)	73 (14%)	
MOF (before PICU admission)	304	36 (50%)	62 (27%)	98 (32%)	<0.001
PARDS (before PICU admission)	226	19 (36%)	23 (13%)	42 (19%)	<0.001
POPC at PICU admission	491				0.005
1		24 (28%)	188 (46%)	212 (43%)	
2		30 (35%)	142 (35%)	172 (35%)	
3		14 (16%)	34 (8.4%)	48 (9.8%)	
4		15 (18%)	38 (9.4%)	53 (11%)	
5		2 (2.4%)	4 (1.0%)	6 (1.2%)	
Advanced treatments before PICU admission	512	50 (53%)	109 (26%)	159 (31%)	<0.001
PIM-3 (at PICU admission)	458	10 (4, 19)	1 (1, 6)	2 (1, 8)	<0.001
MOF (during PICU stay)	217	33 (63%)	66 (40%)	99 (46%)	0.005
Mechanical ventilation	464	45 (60%)	221 (57%)	266 (57%)	0.689
NIV pre-InV	464	21 (22%)	42 (10%)	63 (14%)	0.002
InV	464	37 (80%)	196 (81%)	233 (50%)	0.930
Duration of overall ventilation (days)	277	6 (2, 16)	2 (1, 6)	2 (1, 7)	<0.001
PELOD-2 score at PICU admission	414	2.2 (0.3–3.5)	0.6 (0.1–0.9)	0.6 (0.1–1.4)	<0.001
PELOD-2 score on the second day	305	2.2 (0.9–5.5)	0.3 (0.1–0.9)	0.3 (0.1–1.4)	<0.001
PELOD-2 score at PICU discharge	414	1 (0–8)	0 (0–1)	0 (0–1)	<0.001
Cardiac arrest	480	15 (19%)	19 (4.7%)	34 (7.1%)	<0.001
TPN	480	44 (56%)	112 (28%)	156 (32%)	<0.001
Infection	512	45 (47%)	120 (29%)	165 (32%)	<0.001
Sepsis	162	19 (42%)	48 (41%)	67 (41%)	0.930
LOS (days)	506	4 (1–16)	3 (1–8)	3 (1–9)	0.248
Surgical patients with LOS<48 h	506	10 (11%)	74 (18%)	84 (16%)	0.085
In-PICU mortality	506	38 (41%)	40 (10%)	78 (15%)	<0.001

Data are presented using frequencies and percentages for categorical variables and medians and interquartile range (IQR) for continuous ones. The column ‘N’ reported the number of non-missing observation for each variable

*Abbreviations*: *HSCT* Hematopoietic stem-cell transplantation, *OE* Onco-haematological, *ALL* Acute lymphoblastic leukaemia, *AML* Acute myeloid leukaemia, *NHL* Non-Hodgkin lymphoma, *HL* Hodgkin lymphoma, *PICU* Paediatric intensive care unit, *MOF* Multiple organ failure, *PARDS* Paediatric acute respiratory distress syndrome, *POPC* Paediatric overall performance category, *PIM* Paediatric index of mortality, *NIV* Non-invasive ventilation, *InV* Invasive ventilation, *PELOD* Paediatric logistic organ dysfunction, *TPN* Total parenteral nutrition, *LOS* Length of stay

### Solid versus non-solid tumors

Comparing patients with solid versus hematologic tumors (Table 5), patients with solid tumors were younger (p 0.001), were admitted from a different hospital (p 0.001), and had not received prior therapy (p 0.001). Additionally, before PICU admission, PARDS was less frequent in patients with solid tumors (p = 0.005), advanced treatments were less used (p 0.001), and PELOD-2 score on admission was lower (p = 0.003). Cardiac arrest (p = 0.001), MOF (p 0.001), TPN use (p 0.001), and sepsis (p 0.001), were less frequent. LOS and MV were shorter (p 0.001). PIM-3 (p 0.001), PELOD-2 scores on day 2 and at discharge (p0.001) and in-PICU mortality were lower (6.2% vs 25%, respectively; p0.001).

**Table 5:** Sample characteristics according to tumor type (solid/non-solid)

**Characteristic**	***N***	**Solid tumor**, *N* = 262	**Non-solid tumor**, *N* = 252	**Overall**, *N* = 514	***p*-value**
Age (years) Treatment phase	510 508	5.8 (2.0–11.4)	9.3 (3.6–13.0)	7.2 (2.6–12.6)	<0.001 <0.001
On therapy		82 (32%)	151 (61%)	233 (46%)	
Out of therapy		46 (18%)	45 (18%)	91 (18%)	
Not yet started therapy		131 (51%)	53 (21%)	184 (36%)	
Admission from	510				<0.001
Unit of the same hospital		137 (53%)	177 (71%)	314 (62%)	
Unit of other hospitals		44 (17%)	25 (10%)	69 (14%)	
Emergency department		24 (9.2%)	19 (7.6%)	43 (8.4%)	
PICU of other hospitals		7 (2.7%)	4 (1.6%)	11 (2.2%)	
Other		48 (18%)	25 (10%)	73 (14%)	
MOF (before PICU admission)	303	14 (12%)	84 (45%)	98 (32%)	<0.001
PARDS (before PICU admission)	225	8 (9.0%)	33 (24%)	41 (18%)	0.005
POPC at PICU admission	491				0.533
1		117 (45%)	96 (41%)	213 (43%)	
2		89 (34%)	82 (35%)	171 (35%)	
3		27 (10%)	21 (9.0%)	48 (9.8%)	
4		23 (8.9%)	30 (13%)	53 (11%)	
5		2 (0.8%)	4 (1.7%)	6 (1.2%)	
Advanced treatments before PICU admission	514	43 (16%)	118 (47%)	161 (31%)	<0.001
PIM-3 (at PICU admission)	457	1 (0–2)	7 (2–18)	2 (1–8)	<0.001
MOF (during PICU stay)	216	18 (22%)	81 (60%)	99 (46%)	<0.001
Mechanical ventilation	463	141 (58%)	125 (57%)	266 (57%)	0.700
NIV pre-InV	463	11 (4.2%)	52 (21%)	63 (14%)	<0.001
InV	463	135 (87%)	98 (74%)	233 (50%)	0.006
Duration of overall ventilation (days)	277	1 (1–4)	5 (2–10)	2 (1–7)	<0.001
PELOD score at PICU admission	413	0.6 (0.1–0.9)	0.6 (0.2–2.2)	0.6 (0.1–1.4)	0.003
PELOD score on the second day	304	0.1 (0.1–0.6)	0.9 (0.2–3.5)	0.3 (0.1–1.4)	<0.001
PELOD score at PICU discharge	413	0 (0–0)	0 (0–1)	0 (0–1)	<0.001
Cardiac arrest	479	9 (3.5%)	25 (11%)	34 (7.1%)	0.001
TPN	479	47 (19%)	109 (48%)	156 (33%)	<0.001
Infection	514	37 (14%)	128 (51%)	165 (32%)	<0.001
Sepsis	161	4 (12%)	62 (49%)	66 (41%)	<0.001
LOS (days)	505	2 (1–6)	4 (2–12)	3 (1–9)	<0.001
Surgical patients with LOS<48 h	505	75 (29%)	9 (4%)	84 (16%)	<0.001
In-PICU mortality	506	16 (6.2%)	62 (25%)	78 (15%)	<0.001

Data are presented using frequencies and percentages for categorical variables and medians and interquartile range (IQR) for continuous ones. The column ‘N’ reported the number of non-missing observation for each variable

*Abbreviations*: *HSCT* Hematopoietic stem-cell transplantation, *PICU* Paediatric intensive care unit, *MOF* Multiple organ failure, *PARDS* Paediatric acute respiratory distress syndrome, *POPC* Paediatric overall performance category, *PIM* Paediatric index of mortality, *NIV* Non-invasive ventilation, *InV* Invasive ventilation, *PELOD* Paediatric logistic organ dysfunction, *TPN* Total parenteral nutrition, *LOS* Length of stay

### Risk factors analysis

The results of univariate regression analysis for the main outcomes are reported in Table 6 and 7. Before PICU admission non-solid tumor, HSCT, MOF and PARDS, PIM-3 and POPC were independent predictors of both in-PICU mortality and longer PICU LOS. During PICU stay MOF, NIV and pre-intubation NIV, TPN and the occurrence of infections were also statistically significant predictors of mortality and longer PICU stay.

**Table 6:** Univariate logistic regression for in-PICU mortality

*Predictors*	*OR (95% CI)*	*p*
***Pre-PICU***		
Sex (male)	0.97 (0.59; 1.57)	0.896
Age (in years)	1.06 (1.02; 1.11)	0.008
Non-solid tumor	4.99 (2.85; 9.21)	<0.001
Treatment phase	0.63 (0.47; 0.84)	0.002
HSCT	6.61 (3.90; 11.26)	< 0.001
MOF before PICU admission	5.14 (2.89; 9.31)	< 0.001
Respiratory failure	3.44 (2.09; 5.72)	< 0.001
Hemodynamic failure	7.32 (4.28; 12.60)	< 0.001
Acute Kidney Injury	5.88 (3.28; 10.50)	< 0.001
PARDS before PICU admission	3.93 (1.86; 8.26)	< 0.001
POPC at PICU admission	2.01 (1.60; 2.55)	< 0.001
PIM3 at PICU admission	1.06 (1.04; 1.08)	< 0.001
***In-PICU***		
Priority of admission (1 & 2 vs 3, 4a, 4b)	0.16 (0.07; 0.39)	< 0.001
PARDS during PICU stay	1.39 (0.65; 2.90)	0.380
MOF during PICU stay	7.70 (3.72; 17.31)	< 0.001
Duration of mechanical ventilation	1.02 (1.00; 1.04)	0.088
Mechanical ventilation	1.81 (0.49; 11.72)	0.437
NIV	2.71 (1.45; 5.08)	0.002
NIV pre-InV	4.47 (2.33; 8.64)	< 0.001
InV	0.97 (0.47; 2.18)	0.943
Cardiac arrest	220.80 (62.93; 1404.37)	< 0.001
TPN	1,81 (0.49; 11.72)	< 0.001
Infection	2.54 (1.55; 4.18)	<0.001
Sepsis	2.35 (1.13; 5.01)	0.024
LOS	1.00 (1.00; 1.01)	0.163
Surgery with LOS less than 48 h	0.00 (0.00; 8248.25)	0.981

*Abbreviations:* *OR *Odds ratio, *CI* Confidence interval, *PARDS* Paediatric acute respiratory distress syndrome, *PICU* Paediatric intensive care unit, *POPC* Paediatric overall performance category, *PIM* Paediatric index of mortality, *NIV* Non-invasive ventilation, *InV* Invasive ventilation, *TPN* Total parenteral nutrition, *LOS* Length of stay

**Table 7:** Univariate Zero-adjusted Gamma model for PICU LOS

*Predictors*	*Exp[beta] (95% CI)^*	*p*	*Estimate (95% CI)*	*p*
***Pre-PICU***				
Sex (male)	0.99 (0.82; 1.20)	0.924	0.40 (−1.50; 2.30)	0.679
Age (in years)	0.99 (0.98; 1.02)	0.933	0.01 (−0.16; 0.17)	0.946
Non-solid tumor	1.83 (1,53; 2.20)	<0.001	2.66 (0.76; 4.56)	0.006
Treatment phase	0.93 (0.84; 1.03)	0.159	−0.33 (−1.39; 0.72)	0.536
HSCT	1.48 (1.53; 1.91)	0.002	2.07 (−0.35; 4.49)	0.094
MOF before PICU admission	1.67 (1.30; 2.14)	<0.001	3.76 (1.11; 6.41)	0.005
Respiratory failure	2.20 (1.83; 2–65)	<0.001	5.64 (3.78; 7.50)	<0.001
Hemodynamic failure	1.93 (1.51; 2.47)	<0.001	3.98 (1.64; 6.31)	0.001
Acute Kidney Injury	2.37 (1.81; 3.12)	<0.001	7.24 (4.63; 9.85)	<0.001
PARDS before PICU admission	1.78 (1.23; 2.57)	0.003	4.85 (0.77; 8.93)	0.020
POPC at PICU admission	1.11 (1.01; 1.22)	0.026	0.50 (−0.45; 1.45)	0.303
PIM3 at PICU admission	1.02 (1.01; 1.03)	<0.001	0.08 (0.02; 0.14)	0.008
***In-PICU***				
Priority of admission (1 & 2 vs 3, 4a, 4b)	1.21 (0.76; 1.93)	0.434	2.18 (−2.39; 6.76)	0.349
PARDS during PICU stay	1.49 (1.07; 2.09)	0.020	3.96 (−0.13; 8.06)	0.058
MOF during PICU stay	1.54 (1.17; 2.03)	0.002	3.11 (−0.10; 6.31)	0.058
Duration of mechanical ventilation	1.08 (1.07; 1.11)	<0.001	0.79 (0.66; 0.91)	<0.001
Mechanical ventilation	2.06 (1.26; 3.37)	0.004	0.40 (−1.50; 2.30)	<0.001
NIV	1.87 (1.45; 2.42)	<0.001	4.55 (1.65; 7.45)	0.002
NIV pre-InV	1.80 (1.34; 2.41)	<0.001	0.01 (−0.16; 0.17)	<0.001
InV	1.38 (1.01; 1.88)	0.042	3.34 (−0.21; 6.89)	0.065
Cardiac arrest	1.64 (1.11; 2.40)	0.012	2.66 (0.76; 4.56)	0.314
TPN	2.91 (2.42; 3.50)	<0.001	2.07 (−0.35; 4.49)	<0.001
Infection	2.11 (1.74; 2.56)	<0.001	5.09 (3.19; 7.00)	<0.001
Sepsis	0.80 (0.57; 1.11)	0.183	5.09 (3.19; 7.00)	0.082
Surgery with LOS less than 48 h	0.12 (0.10; 1.15)	<0.001		

^Results from Zero-adjusted Gamma (ZAGA) model, where Exp[beta] indicates the mean ratio, the expected variation of LOS: for each unit increment of the predictor, the mean LOS varies by multiplying by a value equal to Exp(beta)

*Abbreviations*: *HSCT* Hematopoietic stem-cell transplantation, *MOF* Multiple organ failure, *PICU* Paediatric intensive care unit, *PARDS* Paediatric acute respiratory distress syndrome, *POPC* Paediatric overall performance category, *PIM* Paediatric index of mortality, *NIV* Non-invasive ventilation, *InV* Invasive ventilation, *TPN* Total parenteral nutrition

Table 8 shows the results of multivariable regression analysis for in-PICU mortality and LOS. Models with the best statistical measures of goodness of fit are reported. Among all pre-PICU risk-factors, receipt of HSCT (OR 3.48, 95%CI 1.5–8.11), POPC (OR 1.72, 95%CI 1.23–2.42) and PIM-3 score at admission (OR 1.03, 95%CI 1.01–1.06) were associated with increased in-PICU mortality. Considering the in-PICU risk-factors the first two priority levels of admission were associated with a lower risk of mortality compared to priority levels 3, 4a and 4b (OR 0.15, 95%CI 0.04–0.51) whereas the presence of MOF and cardiac arrest (OR 4.83, 95%CI 1.66–15.71 and OR 82.16, 95%CI 14.19–1594.61, respectively), were associated with an increased risk of in-PICU mortality (Table 8, panel A).

**Table 8:** Multivariable logistic and Zero-adjusted Gamma regression models

***Panel A. Outcome: in-PICU mortality***	***OR (95% CI)***	***p***
Pre-PICU predictors*		
Age	1.06 (0.98–1.15)	0.168
Non-solid tumor	1.47 (0.57–3.95)	0.433
HSCT	3.48 (1.50–8.11)	0.004
MOF before PICU admission	1.74 (0.71–4.22)	0.223
POPC at PICU admission	1.72 (1.23–2.42)	0.001
PIM3 at PICU admission	1.03 (1.01–1.06)	0.005
In-PICU predictors**		
Priority of admission (1 & 2 vs 3, 4a, 4b)	0.15 (0.04–0.51)	0.003
MOF during PICU stay	4.83 (1.66–15.71)	0.005
Mechanical ventilation	0.83 (0.10–12.82)	0.878
TPN	0.72 (0.24–2.09)	0.543
Cardiac arrest	82.16 (14.19–1594.61)	<0.001
Infection	2.05 (0.71–6.33)	0.194
***Panel B. Outcome: in-PICU LOS***	***Exp[μ] (95% CI)^***	***p***
*Pre-PICU predictors §*		
Non-solid tumor	0.77 (0.52–1.13)	0.185
PARDS before PICU admission	3.09 (1.84–5.17)	<0.001
Acute Kidney Injury	1.68 (1.07—2.64)	0.024
POPC at PICU admission	1.24 (1.06—1.45)	0.007
PIM 3 at PICU admission	1.02 (1.01–1.03)	<0.001
Priority of admission (1 & 2 vs 3, 4a, 4b)	0.61 (0.28–1.31)	0.207
*In-PICU predictors* ^*§§*^		
Non-solid tumor	1.17 (0.88–1.55)	0.276
Duration of mechanical ventilation (days)	1.07 (1.0 6–1.09)	<0.001
TPN	1.37 (1.02–1.83)	0.036
Infection	1.07 (0.81–1.40)	0.645

*Abbreviations*: *PICU* Paediatric intensive care unit, *HSCT* Hematopoietic stem-cell transplantation, *MOF* Multiple organ failure, *POPC* Paediatric overall performance category, *PIM* Paediatric index of mortality, *TPN* Total parenteral nutrition, *PARDS* Paediatric acute respiratory distress syndrome

^*^ N. observations: 259; R^*2*^ *: 0.307*

^**^ *N. observations: 188; R* ^*2*^ *: 0.438*

^*§*^ N. observations: 199; AIC: 1421.89

^*§§*^ N. observations: 274; AIC: 1749.63

^ Results from Zero-adjusted Gamma (ZAGA) model, where Exp[μ] indicates the expected variation of LOS

PARDS before PICU admission and the presence of acute kidney injury were pre-PICU risk factors associated with an increased risk of prolonged LOS (Exp(beta) = 3.09, 95%CI 1.84–5.17 and Exp(beta) = 1.68, 95%CI 1.07–2.64, respectively), along with PIM 3 (Exp(beta) = 1.24, 95%CI 1.06–1.45) and POPC at admission (Exp(beta) = 1.02, 95%CI 1.01–1.03). Considering the in-PICU variables, TPN use and total duration of MV (both NIV and InV) were associated with a higher risk of prolonged LOS (Exp(beta) 1.37, 95%CI 1.02–1.83 and Exp(beta) 1.07, 95%CI 1.06–1.09, respectively) (Table 8, panel B).

Table 9 shows the results of the multivariable regression analysis for in-PICU mortality and LOS, excluding surgical patients admitted for less than 48 h. This analysis reported the same risk factors except for the POPC score on admission.

**Table 9:** Multivariable logistic and Zero-adjusted Gamma regression models excluding post-surgical patients with a PICU LOS<48 h

***Panel A. Outcome: in-PICU mortality***	***OR (95% CI)***	***p***
Pre-PICU predictors*		
Age	1.04 (0.96–1.12)	0.316
Non-solid tumor	1.15 (0.47–2.91)	0.758
HSCT	3.44 (1.54–7.76)	0.003
MOF before PICU admission	2.14 (0.93–4.91)	0.072
POPC at PICU admission	1.87 (1.36–2.59)	<0.001
PIM3 at PICU admission	1.02 (1.01–1.05)	0.024
In-PICU predictors**		
Priority of admission (1 & 2 vs 3, 4a, 4b)	0.06 (0.01–0.38)	0.005
MOF during PICU stay	5.48 (1.74–20.06)	0.006
Mechanical ventilation	0.55 (0.05–9.42)	0.633
TPN	0.71 (0.22–2.23)	0.559
Cardiac arrest	74.01 (12.41–1457.23)	<0.001
Infection	1.48 (0.48–4.80)	0.503
***Panel B. Outcome: in-PICU LOS***	***Exp[μ] (95% CI)^***	***p***
*Pre-PICU predictors §*		
Non-solid tumor	0.86 (0.63–1.16)	0.318
PARDS before PICU admission	1.73 (1.17–2.56)	0.007
Acute Kidney Injury	2.05 (1.42—2.96)	<0.001
POPC at PICU admission	1.05 (0.91—1.20)	0.542
PIM 3 at PICU admission	1.01 (1.00–1.03)	0.012
Priority of admission (1 & 2 vs 3, 4a, 4b)	1.27 (0.68–2.37)	0.454
*In-PICU predictors*^*§§*^		
Non-solid tumor	0.99 (0.78–1.27)	0.997
Duration of mechanical ventilation (days)	1.05 (1.04–1.07)	< 0.001
TPN	1.67 (1.31–1.42)	0.036
Infection	1.18 (0.94–1.48)	0.144

*Abbreviations: PICU* Paediatric intensive care unit, *HSCT* Hematopoietic stem-cell transplantation, *LOS* Length of stay. *MOF* Multiple organ failure, *POPC* Paediatric overall performance category, *PIM* Paediatric index of mortality, *TPN* Total parenteral nutrition, *PARDS* Paediatric acute respiratory distress syndrome

^*^ *N. observations: 240; R* ^*2*^ *: 0.259*

^**^ *N. observations: 131; R* ^*2*^ *: 0.476*

^*§*^ N. observations: 170; AIC: 1110.54

^*§§*^ N. observations: 225; AIC: 1396.22

^ Results from Zero-adjusted Gamma (ZAGA) model, where Exp [μ] indicates the expected variation of LOS

## Discussion

Our study evaluated a national cohort of pediatric cancer patients admitted to 14 Italian PICUs from 2019 to 2022. This cohort included patients with a variety of oncohematologic diseases, and showed an overall PICU mortality of 15%, consistent with previously published data [1, 2].

As a main result of our study, the use of MV (both NIV and InV) was not significantly associated with mortality, an inconsistent finding compared to many studies [1, 9–13, 17, 18, 29]. Moreover, the duration of MV was associated with prolonged PICU stay, but not with an increased risk of death. The role of ventilation in pediatric cancer patients is emerging as a key factor. Many authors have tried to understand whether InV may contribute to higher mortality rates or whether the delayed PICU admission and delayed intubation, especially to avoid the increased risk of infection and complications, may result in a condition so severe that survival is compromised. Previous studies have shown poor outcomes for mechanically ventilated pediatric cancer patients [7, 8, 14, 15, 17] with survival rates ranging from 25% [13] to 42.5% [14] to 48% [3], to 58% [30]. However, it is possible that patients with severe respiratory failure receive the MV and are transferred to the PICU too late. Indeed, it is difficult to define the ideal timing of intubation and ventilation in cancer patients. A large study reported that delayed intubation after PICU admission may be associated with an increased risk of mortality in HSCT children: patients who were intubated after 4 days of PICU stay had higher mortality rates [31]. Furthermore, higher mortality rates were reported in children who received NIV prior to intubation [31]. The same results were observed in adult cancer studies, confirming that the time from ICU admission to intubation strongly predicts outcome [32]. Early intubation in this population was reported to improve survival [33]. Our data seem to be consistent with the message of using MV without thinking that it may directly affect mortality [3].

In addition, excluding surgical patients admitted for less than 48 h did not change these results, which adds further consistency to our findings.

Importantly, the presence of PARDS at the time of PICU admission was a predictor of longer PICU LOS but not of mortality. This result seems to support the hypothesis that the severity of respiratory failure may not be the main factor, but the approach to it.

Considering other risk factors, the history of HSCT formed a specific subcohort of patients with higher risk of mortality, as reported in other studies [7–10]. In addition to higher mortality, HSCT patients showed worse clinical and prognostic variables such as higher PELOD-2, PIM-3, and POPC scores, and more frequent MOF. They were also more likely to be in the acute phase of oncologic treatment prior to PICU admission and more likely to be treated with advanced therapy. During PICU admission, they received NIV more frequently, required longer MV, and experienced more infections and cardiac arrests. These findings suggest that in clinical practice should consider a lower threshold for PICU admission in HSCT patients to reduced their elevated risk of mortality.

We also grouped patients with solid tumors (an unusually high percentage given the pediatric age of our sample) versus patients with non-solid (hematologic) tumors. Patients with non-solid tumors had an overall higher risk of complications and worse outcome, including higher risk of mortality, higher PELOD-2 score, PIM-3 and POPC on admission, more frequent MOF, and use of advanced treatments before PICU admission. There were also more likely to be in the acute phase of treatment, require longer MV, and have more frequent infections and cardiac arrests. These findings suggest that patients with non-solid tumors are more critically-ill than those with solid tumors, which is consistent with previous research [7, 16].

We identified major risk factors associated with poor outcomes before and during PICU admission. Our data confirmed the PIM-3 score and POPC as predictors of mortality and longer PICU stay in pediatric cancer patients admitted to the PICU. Higher scores were observed in the HSCT group and in patients with non-solid tumors and were associated with mortality, confirming them as high-risk categories [24, 34]. In our study, the analysis of mortality-related factors is strengthened by the small number of surgical patients, especially in the HSCT population. This analysis is also confirmed when surgical patients admitted to the PICU for less than 48 h are excluded.

AKI before PICU admission was associated with longer PICU stay but not with increased mortality. We hypothesize a possible improvement of AKI during PICU stay, therefore its overall effect on mortality may be weaker [35].

During the PICU admission, the presence of MOF and the occurrence of cardiac arrest were associated with worse outcomes, as reported widely in the literature [7–9, 11, 13, 16, 18].

Our study has several strengths. The multicenter design and the extensive case studies ensure a broad representation of the phenomenon. Additionally, our study provided data not only during PICU management but also during the pre-PICU admission phase, providing insights into potential variables that may influence patient outcomes. Early identification of high-risk patients and prediction of their outcomes may help in the decision-making process regarding the timing of PICU admission and the appropriateness of medical interventions. This is of paramount importance in this fragile population.

The main limitation of our study is the partially retrospective design, which impacted the reported missing values for some variables. Moreover, the inclusion of different centers may have resulted in limited homogeneity between centers. Finally, the high proportion of solid tumors reported in the literature as having low disease severity and mortality risk may have impacted some of our results.

## Conclusions

According to our study a history of HSCT or non-solid tumors, higher PIM-3 score and POPC at admission, presence of MOF and history of cardiac arrest are confirmed factors associated with mortality. The use of MV does not appear to be associated with increased mortality in this population.

## Data Availability

We will include individual deidentified participant data underlying the results reported in this article, after deidentification (text, tables, figures). The data will be available immediately after publication for 5 years after publication to researchers who submit a methodologically sound proposal. Proposals should be sent to angela.amigoni@aopd.veneto.it To gain access, data requesters must sign a data access agreement. (Link will be provided).

## Abbreviations

AKI: Acute kidney injury
CRRT: Continuous renal replacement therapy
ECMO: Extracorporeal membrane oxygenation
HFNC: High-flow nasal cannula
HSCT: Hematopoietic stem cell transplantation
InV: Invasive ventilation
LOS: Length of stay
MV: Mechanical ventilation
MOF: Multiple organ failure
NIV: Non invasive ventilation
PICU: Pediatric intensive care unit
PIM-3: Pediatric index of mortality 3
POPC: Pediatric overall performance category
PARDS: Pediatric acute respiratory distress syndrome
TPN: Total parenteral nutrition

## References

[1] Wosten-van Asperen RM, Van Gestel JPJ, Van Grotel M, Tschiedel E, Dohna-Schwake C, Valla FV et al (2019) PICU mortality of children with cancer admitted to pediatric intensive care unit a systematic review and meta-analysis. Crit Rev Oncol Hematol 142:153–163 31404827 10.1016/j.critrevonc.2019.07.014

[2] Dalton HJ, Slonim AD, Pollack MM (2003) Multicenter outcome of pediatric oncology patients requiring intensive care. Pediatric Hematol Oncol 20(8):643–649 14578035

[3] van Gestel JPJ, Bierings MB, Dauger S, Dalle JH, Pavlíček P, Sedláček P et al (2014) Outcome of invasive mechanical ventilation after pediatric allogeneic hematopoietic SCT: results from a prospective, multicenter registry. Bone Marrow Transplant 49(10):1287–1292 25068426 10.1038/bmt.2014.147

[4] Hallahan AR, Shaw PJ, Rowell G, O’Connell A, Schell D, Gillis J (2000) Improved outcomes of children with malignancy admitted to a pediatric intensive care unit. Crit Care Med 28(11):3718–3721 11098979 10.1097/00003246-200011000-00030

[5] Hume JR, Gupta S, Steiner ME (2014) Historical outcomes of pediatric hematopoietic stem cell transplantation patients requiring critical care. J Pediatr Intensive Care 3(3):83–90 31214457 10.3233/PIC-14099 PMC6530754

[6] Demaret P, Pettersen G, Hubert P, Teira P, Emeriaud G (2012) The critically-ill pediatric hemato-oncology patient: epidemiology, management, and strategy of transfer to the pediatric intensive care unit. Ann Intensive Care 2(1):14 22691690 10.1186/2110-5820-2-14 PMC3423066

[7] Faraci M, Bagnasco F, Giardino S, Conte M, Micalizzi C, Castagnola E et al (2014) Intensive care unit admission in children with malignant or nonmalignant disease: incidence, outcome, and prognostic factors: a single-center experience. J Pediatr Hematol Oncol 36(7):e403–e409 24276033 10.1097/MPH.0000000000000048

[8] Haase R, Lieser U, Kramm C, Stiefel M, Vilser C, Bernig T et al (2011) Management of oncology patients admitted to the paediatric intensive care unit of a general children’s hospital – a single center analysis. Klin Padiatr 223(3):142–146 21567369 10.1055/s-0031-1275291

[9] Lamas A, Otheo E, Ros P, Vázquez JL, Maldonado MS, Muñoz A et al (2003) Prognosis of child recipients of hematopoietic stem cell transplantation requiring intensive care. Intensive Care Med 29(1):91–96 12528028 10.1007/s00134-002-1549-2

[10] Szmit Z, Kosmider-Zurawska M, Król A, Lobos M, Miśkiewicz-Bujna J, Zielinska M et al (2020) Factors affecting survival in children requiring intensive care after hematopoietic stem cell transplantation. A retrospective single-center study Pediatr Transplant 24(5):e13765 32558076 10.1111/petr.13765

[11] Ben Abraham R, Toren A, Ono N, Weinbroum AA, Vardi A, Barzilay Z et al (2002) Predictors of Outcome in the Pediatric Intensive Care Units of Children With Malignancies. J Pediatr Hematol Oncol 24(1):23–26 11902734 10.1097/00043426-200201000-00007

[12] Tamburro RF, Barfield RC, Shaffer ML, Rajasekaran S, Woodard P, Morrison RR et al (2008) Changes in outcomes (1996–2004) for pediatric oncology and hematopoietic stem cell transplant patients requiring invasive mechanical ventilation. Pediatr Crit Care Med 9(3):270–277 18446105 10.1097/PCC.0b013e31816c7260

[13] Aspesberro F, Guthrie KA, Woolfrey AE, Brogan TV, Roberts JS (2014) Outcome of pediatric hematopoietic stem cell transplant recipients requiring mechanical ventilation. J Intensive Care Med 29(1):31–37 22904208 10.1177/0885066612457343

[14] Zinter MS, Dvorak CC, Spicer A, Cowan MJ, Sapru A (2015) New Insights Into Multicenter PICU Mortality Among Pediatric Hematopoietic Stem Cell Transplant Patients. Crit Care Med 43(9):1986–1994 26035280 10.1097/CCM.0000000000001085 PMC5253183

[15] Pillon M, Amigoni A, Contin A, Cattelan M, Carraro E, Campagnano E et al (2017) Risk Factors and Outcomes Related to Pediatric Intensive Care Unit Admission after Hematopoietic Stem Cell Transplantation: A Single-Center Experience. Biol Blood Marrow Transplant 23(8):1335–1341 28461212 10.1016/j.bbmt.2017.04.016

[16] Pillon M, Sperotto F, Zattarin E, Cattelan M, Carraro E, Contin AE et al (2019) Predictors of mortality after admission to pediatric intensive care unit in oncohematologic patients without history of hematopoietic stem cell transplantation: A single-center experience. Pediatr Blood Cancer 66(10):e27892 31250548 10.1002/pbc.27892

[17] Pechlaner A, Kropshofer G, Crazzolara R, Hetzer B, Pechlaner R, Cortina G (2022) Mortality of hemato-oncologic patients admitted to a pediatric intensive care unit: a single center experience. Front Pediatr. 10:795158 35903160 10.3389/fped.2022.795158 PMC9315049

[18] Öztürk M, Botan E, Gün E, Baskin AK, İslamoğlu C, Erkol GH et al (2023) The Determining Factors for Outcome of Pediatric Intensive Care Admitted Children After Stem Cell Transplantation. J Pediatr Hematol Oncol 45(6):e768–e772 36706283 10.1097/MPH.0000000000002610

[19] Harris PA, Taylor R, Thielke R, Payne J, Gonzalez N, Conde JG (2009) Research electronic data capture (REDCap)—A metadata-driven methodology and workflow process for providing translational research informatics support. J Biomed Inform 42:377–381 18929686 10.1016/j.jbi.2008.08.010 PMC2700030

[20] Harris PA, Taylor R, Minor BL, Elliott V, Fernandez M, O’Neal L et al (2019) The REDCap consortium: Building an international community of software platform partners. J Biomed Inform 95:103208 31078660 10.1016/j.jbi.2019.103208 PMC7254481

[21] KDIGO-AKI-AKD-Guideline_Scope-of-Work_2023_Final.pdf. Available from: https://kdigo.org/wp-content/uploads/2016/10/KDIGO-AKI-AKD-Guideline_Scope-of-Work_2023. Cited 2023 Aug 29

[22] Emeriaud G, López-Fernández YM, Iyer NP, Bembea MM, Agulnik A, Barbaro RP et al (2023) Executive Summary of the Second International Guidelines for the Diagnosis and Management of Pediatric Acute Respiratory Distress Syndrome (PALICC-2). Pediatr Crit Care Med 24(2):143–168 36661420 10.1097/PCC.0000000000003147 PMC9848214

[23] Matics TJ, Sanchez-Pinto LN (2017) Adaptation and validation of the pediatric sequential organ failure assessment score and evaluation of the sepsis-3 definition in critically ill children. JAMA Pediatr 171(10):e172352 28783810 10.1001/jamapediatrics.2017.2352 PMC6583375

[24] Straney L, Clements A, Parslow RC, Pearson G, Shann F, Alexander J et al (2013) Paediatric Index of Mortality 3: An Updated Model for Predicting Mortality in Pediatric Intensive Care. Pediatr Crit Care Med 14(7):673–681 23863821 10.1097/PCC.0b013e31829760cf

[25] Leteurtre S, Martinot A, Duhamel A, Proulx F, Grandbastien B, Cotting J et al (2003) Validation of the paediatric logistic organ disfunction (PELOD) score: prospective, observational, multicentre study. The Lancet 19:192–197 10.1016/S0140-6736(03)13908-6 12885479

[26] Benjamini Y, Hochberg Y (1995) Controlling the False Discovery Rate: A Practical and Powerful Approach to Multiple Testing. J R Statist Soc 57:289–300

[27] Rigby RA, Stasinopoulos DM (2005) Generalized Additive Models for Location, Scale and Shape. J R Statist Soc 54(3):507–554

[28] R Core Team. R (2023) A Language and Environment for Statistical Computing. R Foundation for Statistical Computing.

[29] Flerlage T, Kimberly F, Qin Y, Agulnik A, Arias AV, Cheng C et al (2023) Mortality risk factors in pediatric onco-critical care patients and machine learning derived early onco-critical care phenotypes in a retrospective cohort. Crit Care Explor. 5(10):e0976 37780176 10.1097/CCE.0000000000000976 PMC10538916

[30] Chima RS, Daniels RC, Kim MO, Li D, Wheeler DS, Davies SM et al (2012) Improved outcomes for stem cell transplant recipients requiring pediatric intensive care. Pediatr Crit Care Med 13(6):e336–e342 22791094 10.1097/PCC.0b013e318253c945

[31] Rowan CM, Gertz SJ, McArthur J, Fitzgerald JC, Nitu ME, Loomis A et al (2016) Invasive Mechanical Ventilation and Mortality in Pediatric Hematopoietic Stem Cell Transplantation: A Multicenter Study. Pediatr Crit Care Med 17(4):294–302 26910477 10.1097/PCC.0000000000000673

[32] Dumas G, Lemiale V, Rathi N, Cortegiani A, Pène F, Bonny V et al (2021) Survival in immunocompromised patients ultimately requiring invasive mechanical ventilation: A pooled individual patient data analysis. Am J Respir Crit Care Med 204(2):187–196 33751920 10.1164/rccm.202009-3575OC

[33] Saillard C, Lambert J, Tramier M, Chow-Chine L, Bisbal M, Servan L et al (2022) High-flow nasal cannula failure in critically ill cancer patients with acute respiratory failure: Moving from avoiding intubation to avoiding delayed intubation. PLoS ONE 17(6):e0270138 35767521 10.1371/journal.pone.0270138 PMC9242496

[34] Pollack MM, Holubkov R, Funai T, Dean JM, Berger JT, Wessel DL et al (2016) The Pediatric Risk of Mortality Score: update 2015. Pediatr Crit Care Med 17(1):2–9 26492059 10.1097/PCC.0000000000000558 PMC5048467

[35] Sanchez-Pinto LN, Goldstein SL, Schneider JB, Khemani RG (2015) Association between progression and improvement of Acute Kidney Injury and mortality in critically ill children. Pediatr Crit Care Med 16(8):703–710 26132741 10.1097/PCC.0000000000000461

